# Advanced AI-assisted panoramic radiograph analysis for periodontal prognostication and alveolar bone loss detection

**DOI:** 10.3389/fdmed.2024.1509361

**Published:** 2025-01-06

**Authors:** Jarupat Jundaeng, Rapeeporn Chamchong, Choosak Nithikathkul

**Affiliations:** ^1^ Ph.D. in Health Science Program, Faculty of Medicine, Mahasarakham University, Mahasarakham, Thailand; ^2^ Tropical Health Innovation Research Unit, Faculty of Medicine, Mahasarakham University, Mahasarakham, Thailand; ^3^ Dental Department, Fang Hospital, Chiang Mai, Thailand; ^4^ Department of Computer Science, Faculty of Informatics, Mahasarakham University, Mahasarakham, Thailand

**Keywords:** deep learning, convolutional neural networks (CNNs), panoramic radiograph analysis, alveolar bone loss assessment, periodontal prognosis, Thai Association of Periodontology

## Abstract

**Background:**

Periodontitis is a chronic inflammatory disease affecting the gingival tissues and supporting structures of the teeth, often leading to tooth loss. The condition begins with the accumulation of dental plaque, which initiates an immune response. Current radiographic methods for assessing alveolar bone loss are subjective, time-consuming, and labor-intensive. This study aims to develop an AI-driven model using Convolutional Neural Networks (CNNs) to accurately assess alveolar bone loss and provide individualized periodontal prognoses from panoramic radiographs.

**Methods:**

A total of 2,000 panoramic radiographs were collected using the same device, based on the periodontal diagnosis codes from the HOSxP Program. Image enhancement techniques were applied, and an AI model based on YOLOv8 was developed to segment teeth, identify the cemento-enamel junction (CEJ), and assess alveolar bone levels. The model quantified bone loss and classified prognoses for each tooth.

**Results:**

The teeth segmentation model achieved 97% accuracy, 90% sensitivity, 96% specificity, and an F1 score of 0.80. The CEJ and bone level segmentation model showed superior results with 98% accuracy, 100% sensitivity, 98% specificity, and an F1 score of 0.90. These findings confirm the models' effectiveness in analyzing panoramic radiographs for periodontal bone loss detection and prognostication.

**Conclusion:**

This AI model offers a state-of-the-art approach for assessing alveolar bone loss and predicting individualized periodontal prognoses. It provides a faster, more accurate, and less labor-intensive alternative to current methods, demonstrating its potential for improving periodontal diagnosis and patient outcomes.

## Introduction

Periodontitis is a chronic inflammatory disease affecting the gingival tissues and supporting structures of the teeth, often leading to tooth loss. The condition begins with the accumulation of dental plaque, which initiates an immune response (1). According to the classic model of periodontal disease development proposed by Page and Kornman (2), periodontitis is a multifactorial disease influenced by genetic and epigenetic factors, along with modifiable factors such as patient behaviors, medications, and environmental conditions. In addition, site-specific factors, including anatomical variations, may predispose certain areas to increased susceptibility to periodontal lesions. Clinically, periodontitis is characterized by an exaggerated yet ineffective inflammatory response in the connective tissues surrounding the teeth. This unresolved inflammation causes progressive damage to the periodontal ligament and alveolar bone, resulting in clinical signs such as deep periodontal pockets, loss of attachment, and, in advanced cases, tooth mobility or loss (3, 4). Recent studies suggest that the onset and progression of periodontitis affect 19% of adults worldwide, impacting over 1 billion people and ranking it as the 11th most prevalent disease (5, 6). This widespread condition poses a significant public health concern, affecting oral function, aesthetics, social equality, and quality of life (7).

Early and accurate detection of periodontitis is essential for effective treatment. To determine the stage and severity of periodontal disease accurately, dentists must conduct extensive diagnostic procedures, thoroughly evaluating each tooth individually. This meticulous approach is necessary due to the varied clinical manifestations of the disease. Currently, diagnostic methods for periodontitis primarily involve clinical assessments and radiographic evaluations (8). Panoramic radiographs are commonly used to assess alveolar bone loss, a key indicator of disease progression. However, the interpretation of these radiographs can be subjective and influenced by the dentist's experience and the quality of the images (9, 10). These challenges underscore the need for more reliable, objective, and efficient diagnostic tools to improve the accuracy and consistency of periodontal disease detection.

Periodontal prognosis involves predicting the likely outcome or course of periodontal disease, particularly regarding disease progression, stability, or response to treatment. This prognosis is based on evaluating various factors, including the severity of the periodontal disease, the patient's systemic health, habits such as smoking, and oral hygiene practices. Prognoses are typically classified as favorable, questionable, or poor, depending on the likelihood of maintaining periodontal health or halting disease progression (11). However, due to the complexity of periodontal disease and factors such as systemic conditions and dentist expertise, there is no universally accepted standard for periodontal prognosis (12). The most widely used prognostic system, proposed by McGuire and Nunn in 1996 (11), categorizes prognosis into five groups: good, fair, poor, questionable, and hopeless. These categories are based on factors such as disease control, attachment loss, furcation involvement, crown-to-root ratio, and tooth mobility. Additionally, the Thai Association of Periodontology has developed its own classification for periodontal prognosis, which provides clearer assessments of bone support percentages, as detailed in Table 1 (13). These prognostic categories are essential for treatment planning, helping dentists decide on the management of periodontal disease and whether to retain or extract affected teeth.

**Table 1:** Periodontal prognosis as defined by the Thai association of periodontology (13).

Prognostic level	Bone support (from the most severe site)	Probing depth	Mobility	Furcation involvement
Good	>75%	<6 mm	0	0
Fair	50%–75%	<6 mm	0–1	0–1
Poor	50%–75%	≥6 mm	0–2	0–2 (B,Li)
Questionable	25%–50%	≥6 mm	0–3	2–3
Hopeless	<25%	≥6 mm	2–3	3

Recent advancements in artificial intelligence (AI) have brought transformative changes to various fields of medicine, including dentistry (14). AI technologies have demonstrated remarkable potential in automating and enhancing diagnostic processes by providing rapid and accurate assessments of medical images (15, 16). These AI models are designed to overcome the limitations of current methods, offering automated, precise, and consistent diagnostic evaluations (16).

Moreover, in dentistry, AI focuses on caries, periodontal diseases, endodontic lesions, and jawbone pathologies (17, 18). Convolutional Neural Networks (CNNs) have been successful with both two-dimensional (2D) and three-dimensional (3D) images (19, 20). While 3D evaluations are common in implantology, surgery, endodontics, and orthodontics, periodontology mainly uses them to assess furcations, craters, bone defects, root form, and alveolar relationships (21). Standard periodontal assessments typically use periapical, bite-wing, and panoramic radiography for cost-effective, quick, and low-radiation evaluations of alveolar bone levels (22). Additionally, many existing AI models primarily focus on detecting periodontitis without offering a comprehensive approach to assess both bone loss and prognosis (23). In contrast, our study introduces a novel AI-driven approach that utilizes both teeth segmentation and precise analysis of the cemento-enamel junction (CEJ) and alveolar bone levels, significantly improving the detection of alveolar bone loss and prognostication. This model may achieve superior precision and recall metrics. These innovations address the shortcomings of earlier studies, providing more reliable and individualized assessments, which could have significant implications for clinical practice and patient outcomes. These issues have led to the development of supportive diagnostic tools to improve accuracy.

This study aims to develop an innovative AI-driven model using state-of-the-art CNNs to accurately assess the percentage of alveolar bone loss and provide individualized periodontal prognoses based on panoramic radiographs. Additionally, this model will assist dentists in making immediate decisions regarding the management of periodontal disease, including whether to retain or extract affected teeth. It will also facilitate effective communication with patients and support early treatment interventions.

## Materials and methods

### Study design

This retrospective study examined 2,000 panoramic radiographs obtained from the Dental Department of Fang Hospital in Chiang Mai, Thailand. Intraoral examinations were not conducted. The radiographic data were collected between January 2015 and December 2023 using diagnosis codes from the HOSxP Program (Bangkok Medical Software, Bangkok, Thailand). All images were captured with the same device and stored using the SIDEXIS Next Generation Program (Sirona, Bensheim, Germany). To ensure the integrity of the dataset, radiographs were excluded if they exhibited improper patient positioning, poor quality due to movement, uncommon bone morphologies, or if the alveolar bone loss in the affected area could not be accurately assessed. This study was approved by the Ethical Review Board of Fang Hospital (COA No. 03/2566) and the Ethics Committee for Research Involving Human Subjects of Mahasarakham University (No. 533-589/2023). Additionally, the consent letter for data collection for this research project was granted by the director of Fang Hospital in Chiang Mai, Thailand (No. 0033.306/3674).

## Inclusion and exclusion criteria

**Inclusion criteria**:

1. Age: Participants aged 18 years and older to ensure that only fully erupted molars are included, while excluding erupting or unerupted molar teeth.
2. Diagnosis: Individuals diagnosed with periodontitis, as identified through diagnosis codes from the HOSxP Program (Bangkok Medical Software, Bangkok, Thailand).
3. Radiograph Quality: High-quality panoramic radiographs obtained from the SIDEXIS Next Generation Program (Sirona, Bensheim, Germany) and captured using a consistent device.

**Exclusion criteria:**

**Figure 1:**
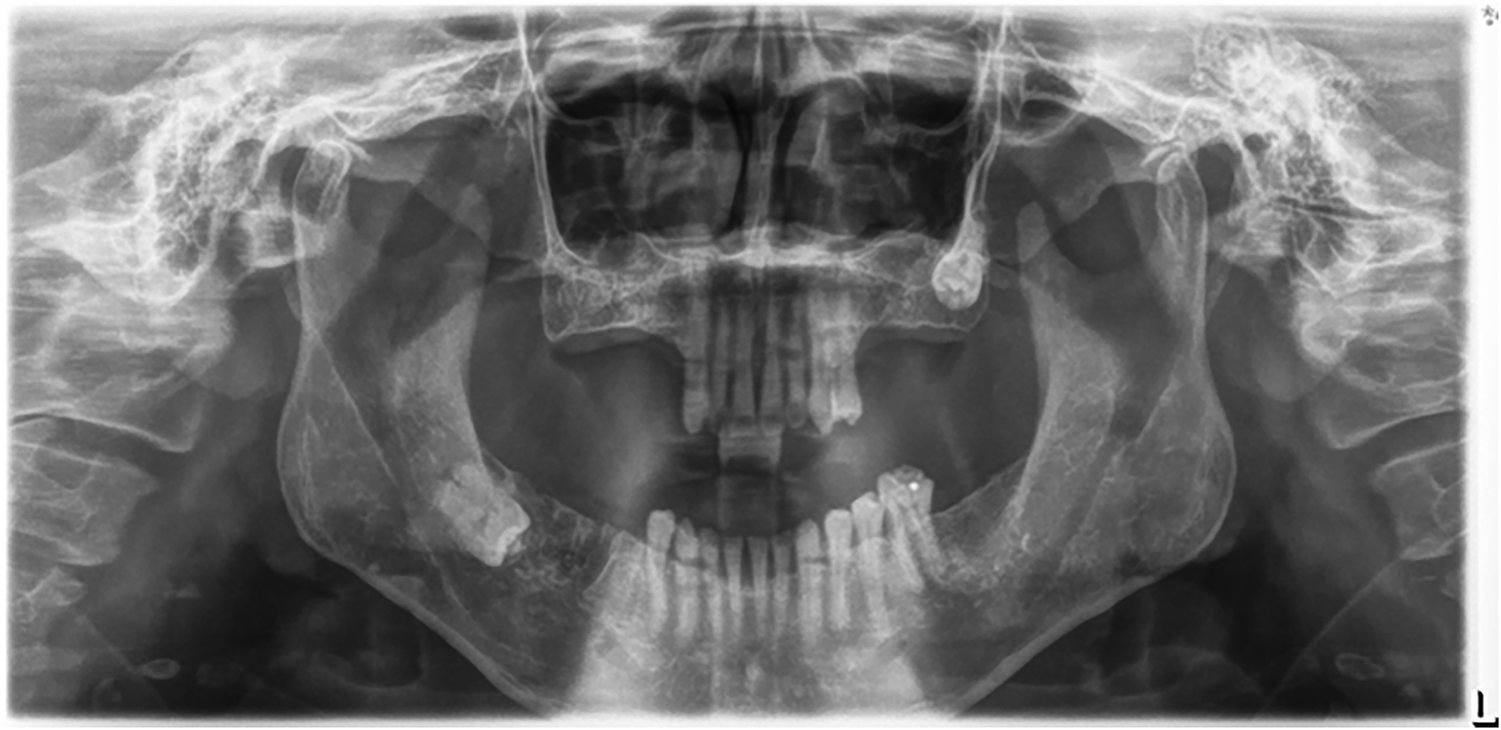
An illustration of a panoramic x-ray image with incorrect patient positioning and a low-quality radiograph.

**Figure 2:**
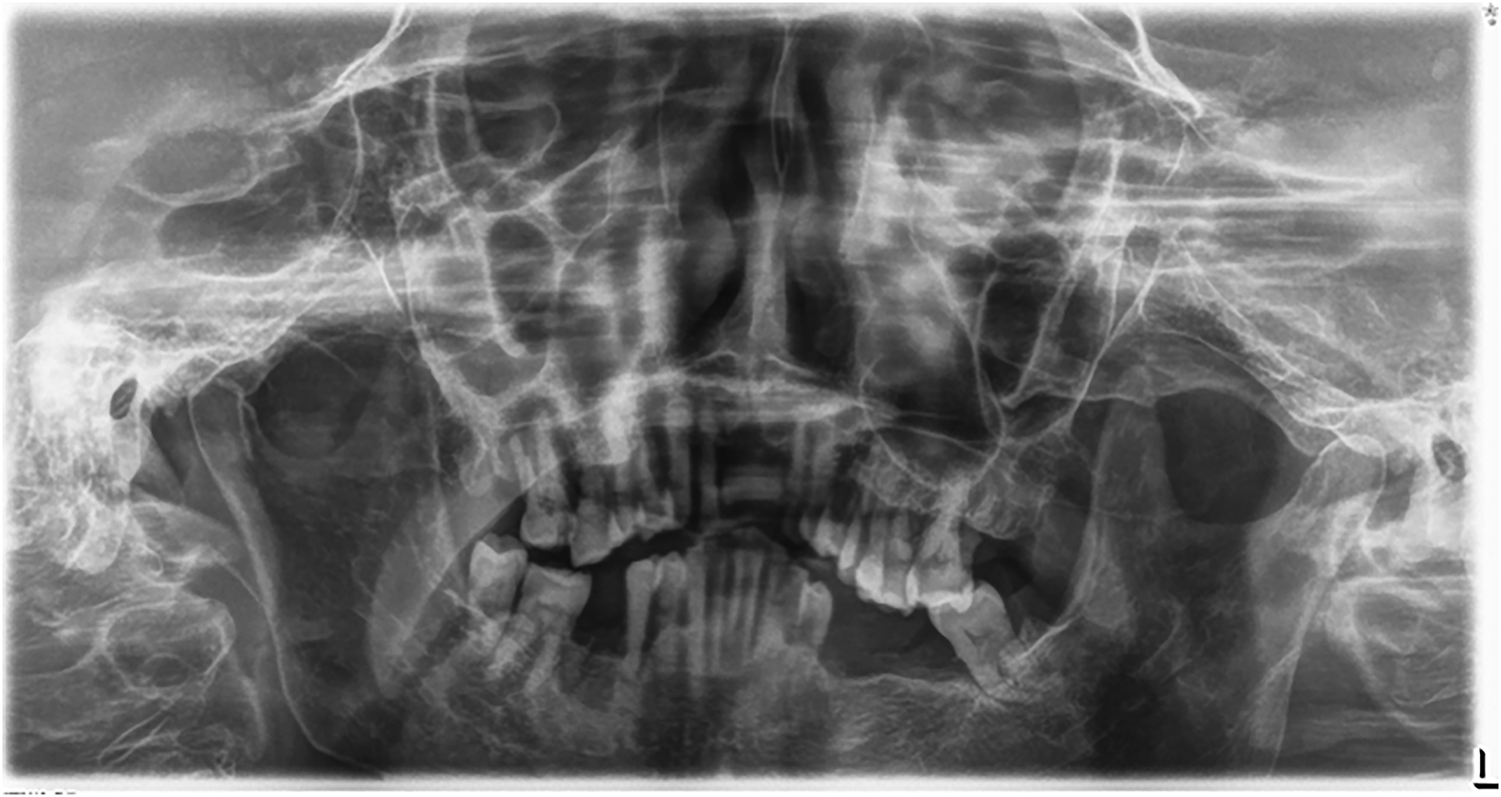
An illustration of panoramic x-ray image where the area could not be accurately selected for determining periodontal bone destruction.

1. Missing Radiographs: Absence of panoramic radiographs in the SIDEXIS Next Generation Program.
2. Image Quality: Radiographs were excluded if they exhibited improper patient positioning, poor quality due to movement, uncommon bone morphologies (Figure 1), or if the alveolar bone loss in the affected area could not be accurately assessed (Figure 2).
3. Panoramic radiographs of patients with craniofacial anomalies, as these conditions may affect bone morphology.

### Data collection

The dataset included 2,000 panoramic radiographs of patients diagnosed with periodontitis, identified using diagnosis codes from the HOSxP Program (Bangkok Medical Software, Bangkok, Thailand). Radiographs were excluded if they showed improper positioning, poor image quality, or uncommon bone morphologies.

### Data handling and ethical considerations

All patient information was anonymized to ensure confidentiality. The study strictly followed ethical guidelines, complying with both institutional and regulatory standards.

### Image enhancement

Image preprocessing involved several enhancement techniques to improve the clarity and quality of the radiographs (24):

1. **Image Sharpening:** Enhanced edges to improve the distinction of pixel boundaries and overall visual interpretability.
2. **Contrast Adjustment:** Utilized histogram equalization to balance brightness levels, making target areas more distinguishable from the background.
3. **Gaussian Filtering:** Applied a 3 × 3 kernel matrix to reduce noise and smooth the images.

After preprocessing, all images were labeled using the LabelMe tool for object segmentation, and the Labelme2yolo tool was employed for data conversion (25). The annotations focused on identifying the CEJ and the alveolar bone level.

### AI model development

**Figure 3:**
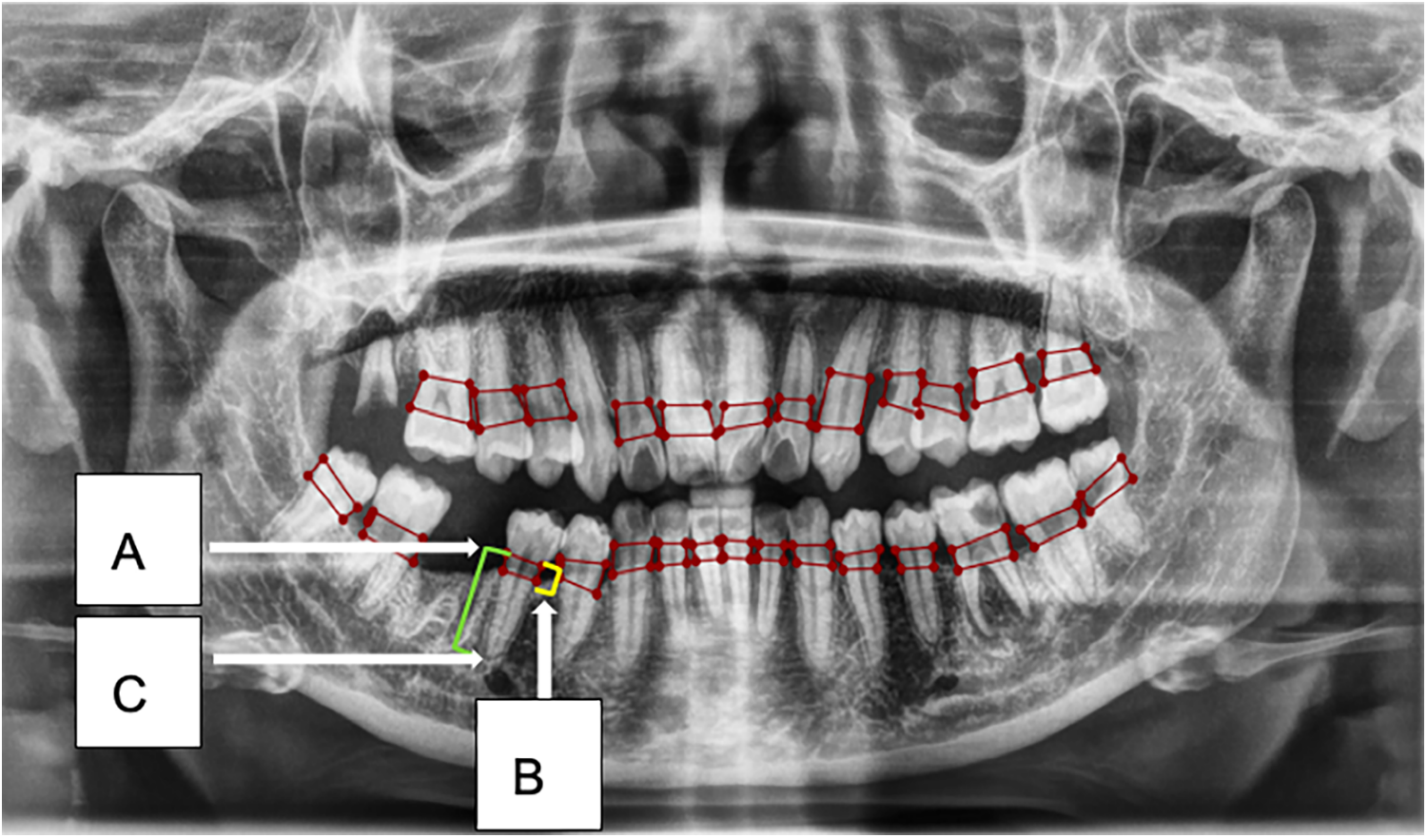
This figure illustrates an example of radiographic bone loss. The color coding for the lines is as follows: yellow indicates the distance from the cemento-enamel junction (CEJ) **(A)** in relation to the alveolar bone crest level **(B)**, green represents the distance from the CEJ to the root apex **(C)**, and red boxes denote the distance from the CEJ to the alveolar bone crest level.

The Convolutional Neural Network (CNN) model, utilizing YOLOv8 (You Only Look Once version 8), was developed to extract regions of interest from the radiographs (26, 27) (Figure 3). YOLOv8 is a state-of-the-art, real-time object detection model widely used in computer vision tasks, including image segmentation and classification. It processes the image in a single pass, which provides both high speed and accuracy, making it particularly suitable for detecting periodontal disease in dental radiographs (28). Additionally, the model's performance was evaluated using mAP50 (mean Average Precision at a 50% Intersection over Union threshold), a metric that calculates the mean precision of predicted bounding boxes where the predicted and true boxes overlap by at least 50%. A higher mAP50 value indicates better model performance in accurately detecting the objects of interest (29).

**Figure 4:**
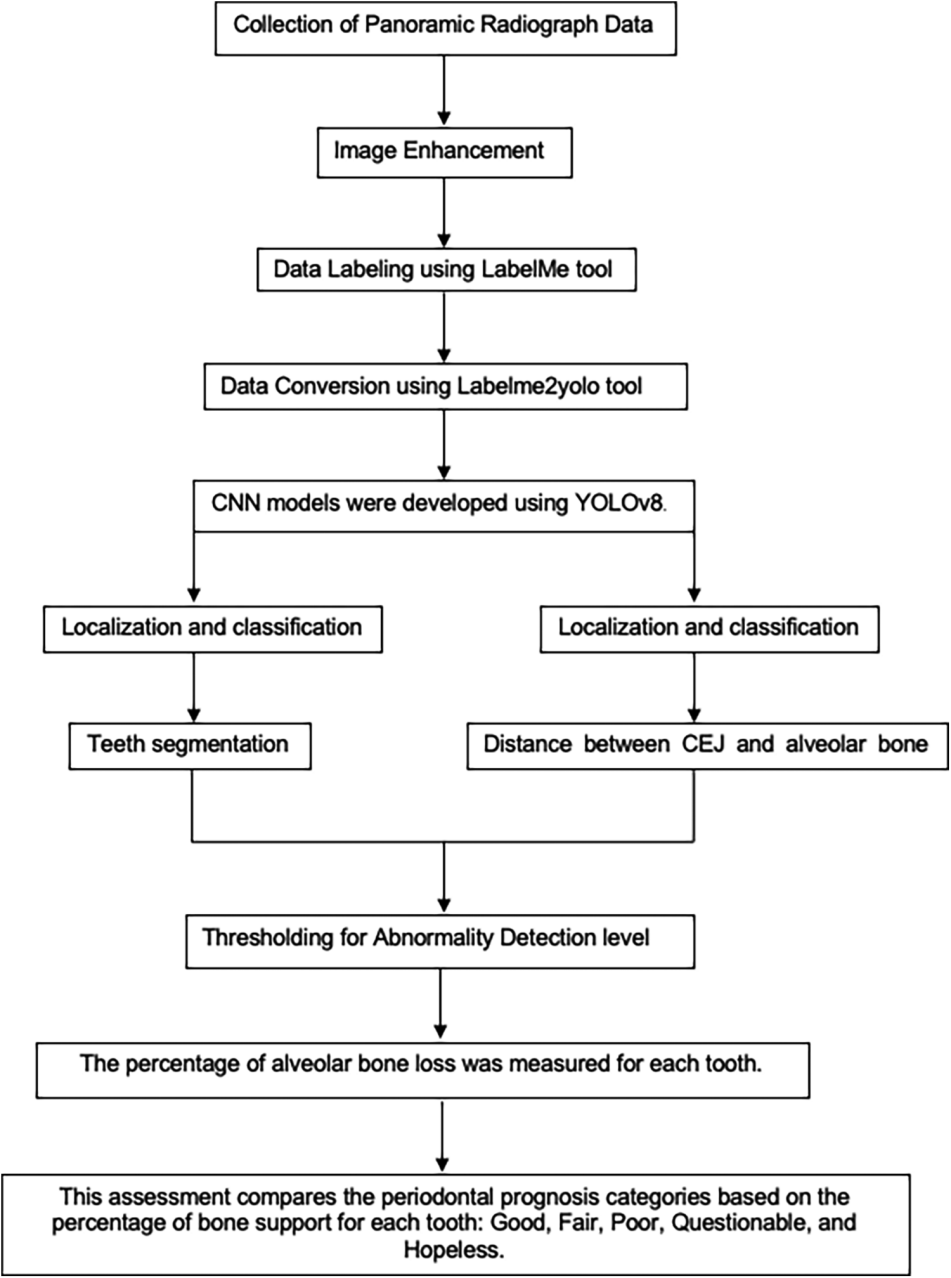
This diagram summarizes the development of the AI model.

The development workflow included:

1. **Data Segmentation:** The dataset was split into training, validation, and test sets in a ratio of 70:10:20.
2. **Training Environment:** The model was trained on a system equipped with an Intel Core i7–8,700 K CPU, 16 GB RAM, Nvidia GeForce RTX2080 GPU with 8 GB of video memory, CUDA Toolkit 9.0, CUDNN V11.7, and Python 3.11.5.
3. **Localization and Classification:** The model identified and localized the areas between the CEJ and the alveolar bone level, generating bounding boxes or heat maps to highlight these regions.
4. **Thresholding for Abnormality Detection:** A thresholding mechanism was applied to evaluate the degree of abnormality by measuring the gap width between the CEJ and the surrounding bone structure (Figure 4). Teeth with gaps exceeding the predefined threshold (e.g., >2 mm) will be flagged as abnormally positioned. To calculate the percentage of bone loss, use the formula:
  $$\mathrm{Percentage}\;\mathrm{of}\;\mathrm{bone}\;\mathrm{loss}=\frac{(\mathrm{CEJ}-\mathrm{Alveolar}\;\mathrm{bone}\;\;\mathrm{rest})-2\;\mathrm{mm}}{(\mathrm{CEJ}-\mathrm{Root}\;\mathrm{apex})-2\;\mathrm{mm}}\times 100$$

### Model evaluation and validation

The accuracy of the model's predictions was evaluated using a confusion matrix, which detailed the rates of True Positives (TP), True Negatives (TN), False Positives (FP), and False Negatives (FN). Key performance metrics, including Precision, Recall, and F1-Score, were calculated to assess the model's efficiency and effectiveness (30, 31).

## Results

The panoramic radiographs used in this study were sourced from the Dental Department of Fang Hospital, Chiang Mai, Thailand. All radiographs were collected from the SIDEXIS Next Generation Program (Sirona, Bensheim, Germany) and which captured from the same device. For each patient, only one radiograph was included in the analysis, resulting in a total dataset of 2,000 images. These images were randomly allocated to the training, validation, and test sets in a 70:10:20 ratio, as detailed in Table 2.

**Table 2:** The demographic data of the patients.

Sex	Numbers of patients	Mean age (years)
Male	823	47.04
Female	1,177	45.27

The primary outcome variables focus on the performance of segmentation tasks critical for diagnosing periodontal disease and assessing prognosis based on radiographic features. Table 3 presents precision measures, with the CEJ and bone level segmentation model outperforming the teeth segmentation model (0.90 vs. 0.80). The F1 Score, reflecting the harmonic mean of precision and sensitivity, was 0.80 for teeth segmentation and 0.90 for the CEJ and bone level segmentation, indicating balanced performance. Sensitivity was perfect (1.0) for the CEJ and bone level model, while the teeth model achieved 0.90. Specificity was 0.96 for the teeth segmentation model and 0.98 for the CEJ and bone level model. Accuracy was higher for the CEJ and bone level model (0.98) compared to the teeth model (0.97), and the Mean Average Precision (mAP50) further emphasized the superiority of the CEJ and bone level model (0.995 vs. 0.92). Overall, the CEJ and bone level segmentation model demonstrated superior performance across all metrics.

**Table 3:** Performance metrics of the two AI models developed for analyzing panoramic radiographs. One model was designed for segmenting teeth, and the other for segmenting the cemento-enamel junction (CEJ) and alveolar bone levels. The models achieved the following scores (32).

	Teeth segmentation model	CEJ and bone level segmentation model
Precision	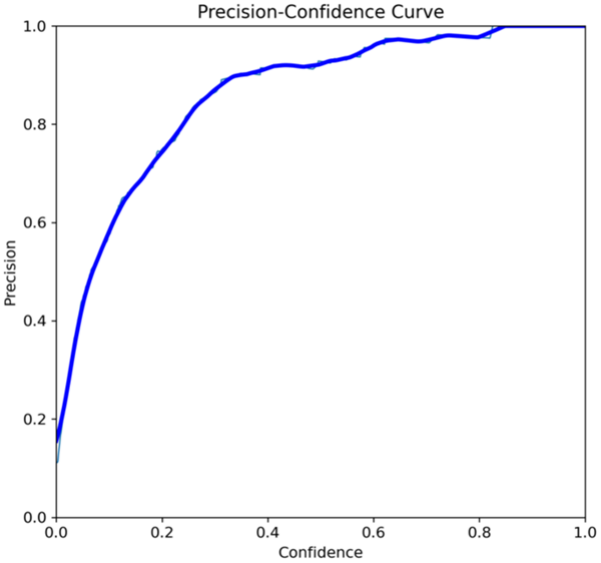 0.80	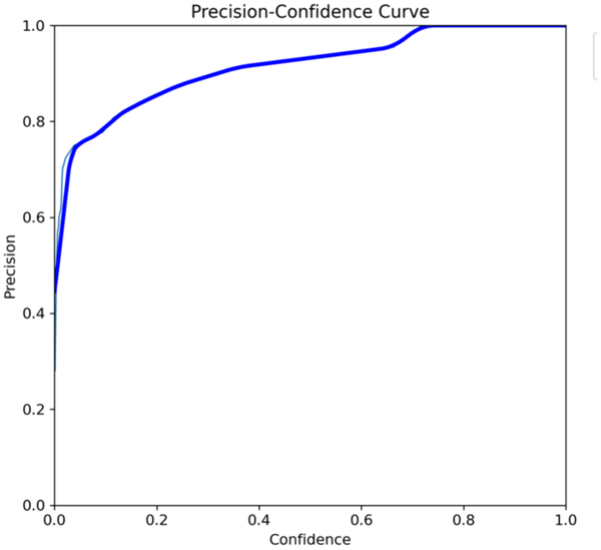 0.90
F1	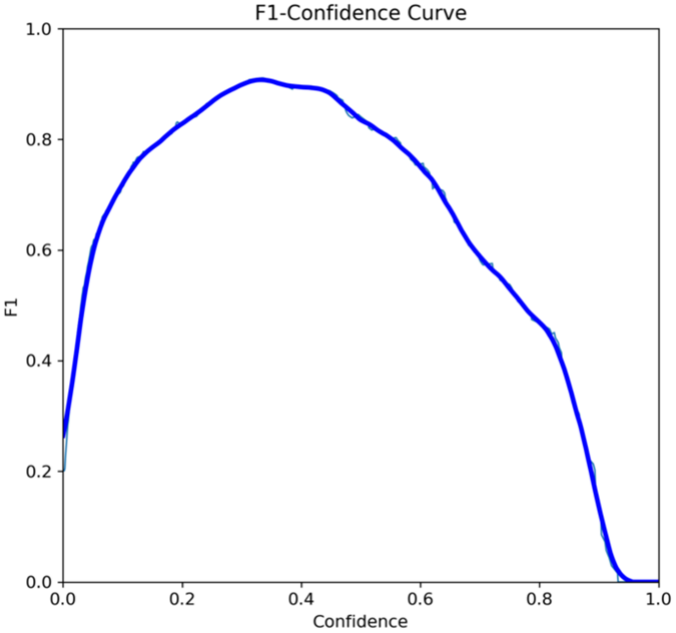 0.80	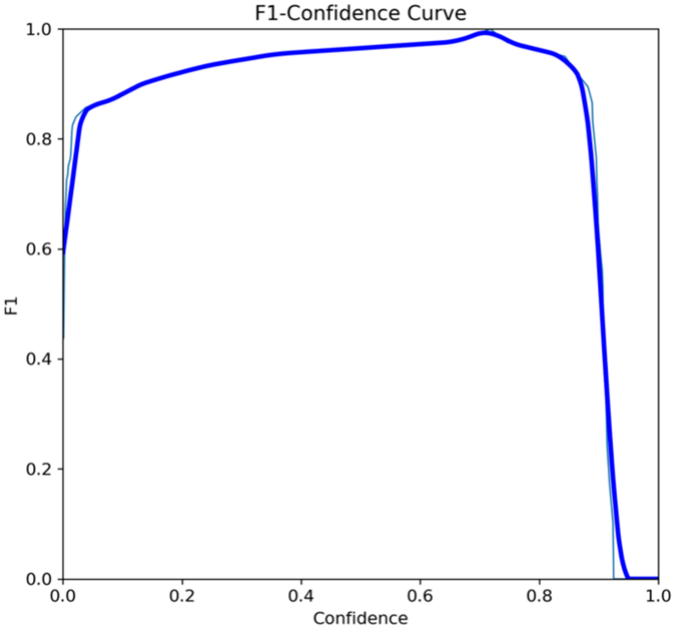 0.90
Sensitivity	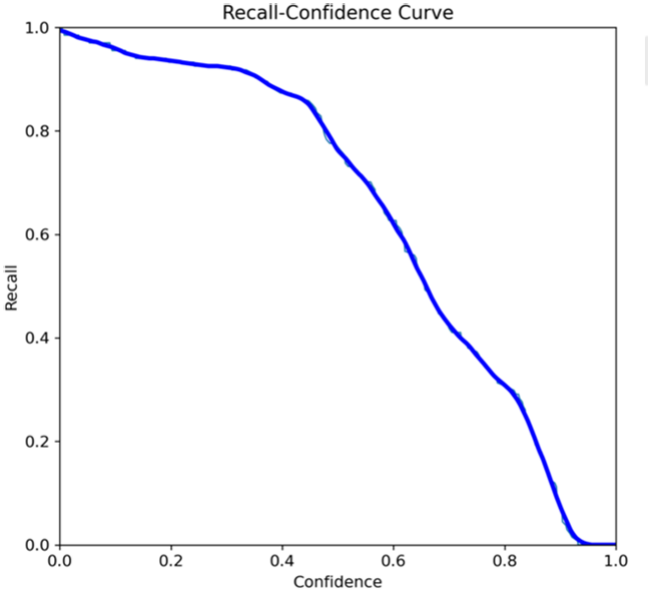 0.90	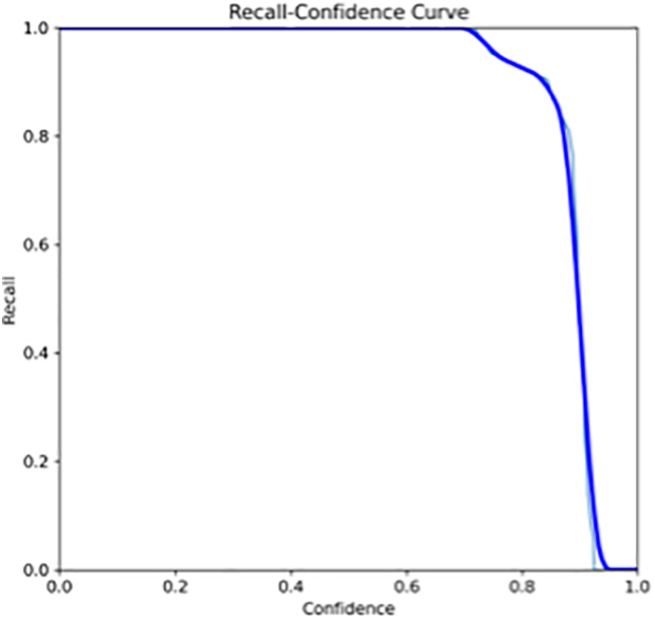 1.0
Specificity	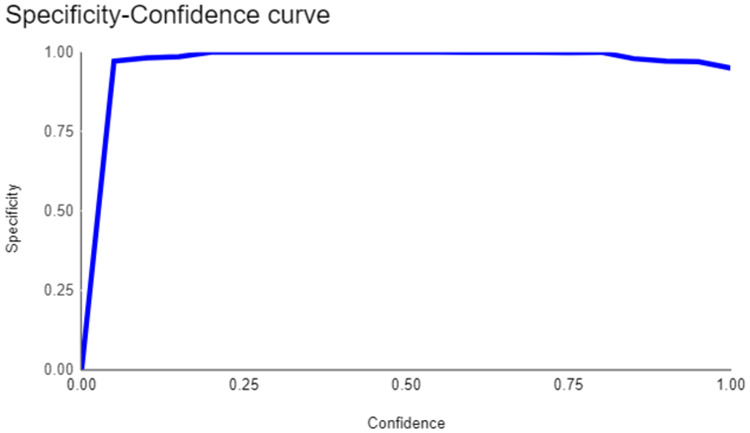 0.96	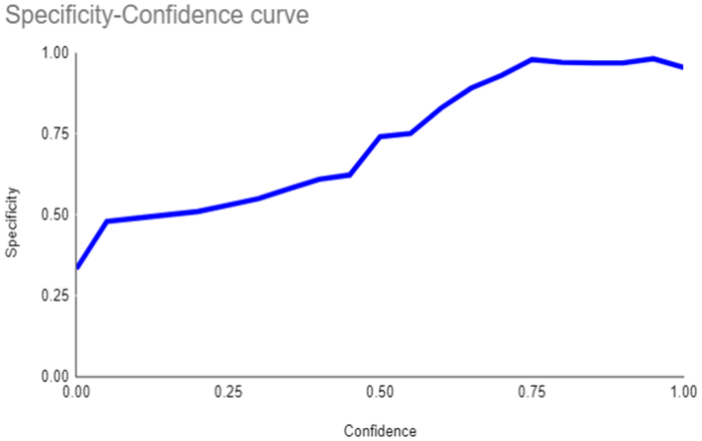 0.98
Accuracy	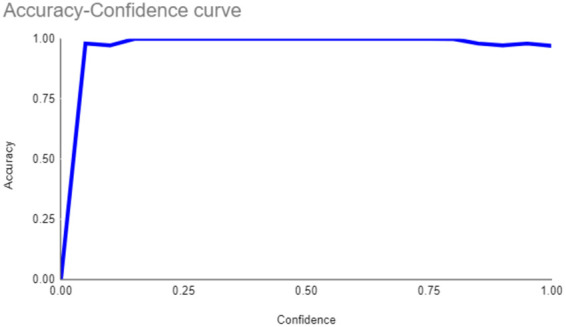 0.97	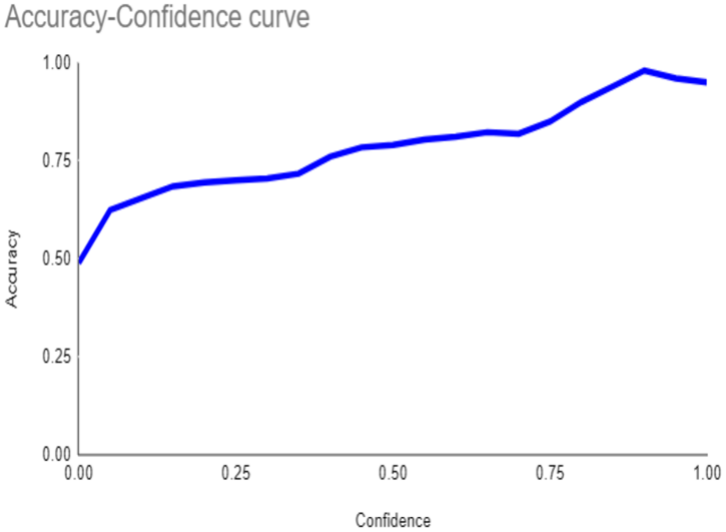 098
mAP50	0.92	0.995

**Accuracy:** The overall percentage of correct predictions made by the AI model compared to the actual diagnosis.

**Sensitivity (Recall):** The ability of the model to correctly identify cases of periodontal disease (true positives).

**Specificity:** The model's ability to correctly identify cases where periodontal disease is absent (true negatives).

**Precision:** The proportion of positive identifications that were actually correct.

**F1 Score:** The harmonic mean of precision and recall, providing a balance between them.

**mAP50 (mean Average Precision at a 50% Intersection over Union threshold):** A standard evaluation metric used in object detection tasks, such as the one used in the AI model for detecting periodontal disease.

Moreover, both the CEJ and bone level segmentation model (Table 4) and the teeth segmentation model (Table 5) demonstrated strong performance in accurately classifying relevant areas in panoramic radiographs. In Table 4, the CEJ and bone level model correctly predicted 18,385 instances, with only 234 false positives, indicating high precision. The model also exhibited strong recall, with minimal false negatives (11). Similarly, the teeth segmentation model (Table 5) performed well, accurately identifying 983 teeth instances and 18,687 true negatives. However, it had a slightly higher false positive rate (589), where non-teeth areas were incorrectly classified as teeth. Despite the higher false positive rate in the teeth model, both models exhibited high accuracy and efficiency in their respective tasks, with low false negative rates and a strong ability to differentiate between positive and negative classes in their predictions.

**Table 4:** Confusion matrix for the CEJ and bone level segmentation model.

	Actual value		
Predicted value		Positive	Negative
	Positive	TP:508	FP:234
	Negative	FN:11	TN:17877

True Positive (TP): Correctly identified areas indicating bone loss.

True Negative (TN): Correctly identified areas without bone loss.

False Positive (FP): Areas incorrectly labeled as having bone loss when none is present.

False Negative (FN): Areas with bone loss that were incorrectly identified as normal.

**Table 5:** Confusion matrix for the teeth segmentation model.

	Actual value		
Predicted value		Positive	Negative
	Positive	TP:983	FP:589
	Negative	FN:11	TN:18687

True Positive (TP): Correctly identified areas indicating bone loss.

True Negative (TN): Correctly identified areas without bone loss.

False Positive (FP): Areas incorrectly labeled as having bone loss when none is present.

False Negative (FN): Areas with bone loss that were incorrectly identified as normal.

**Figure 5:**
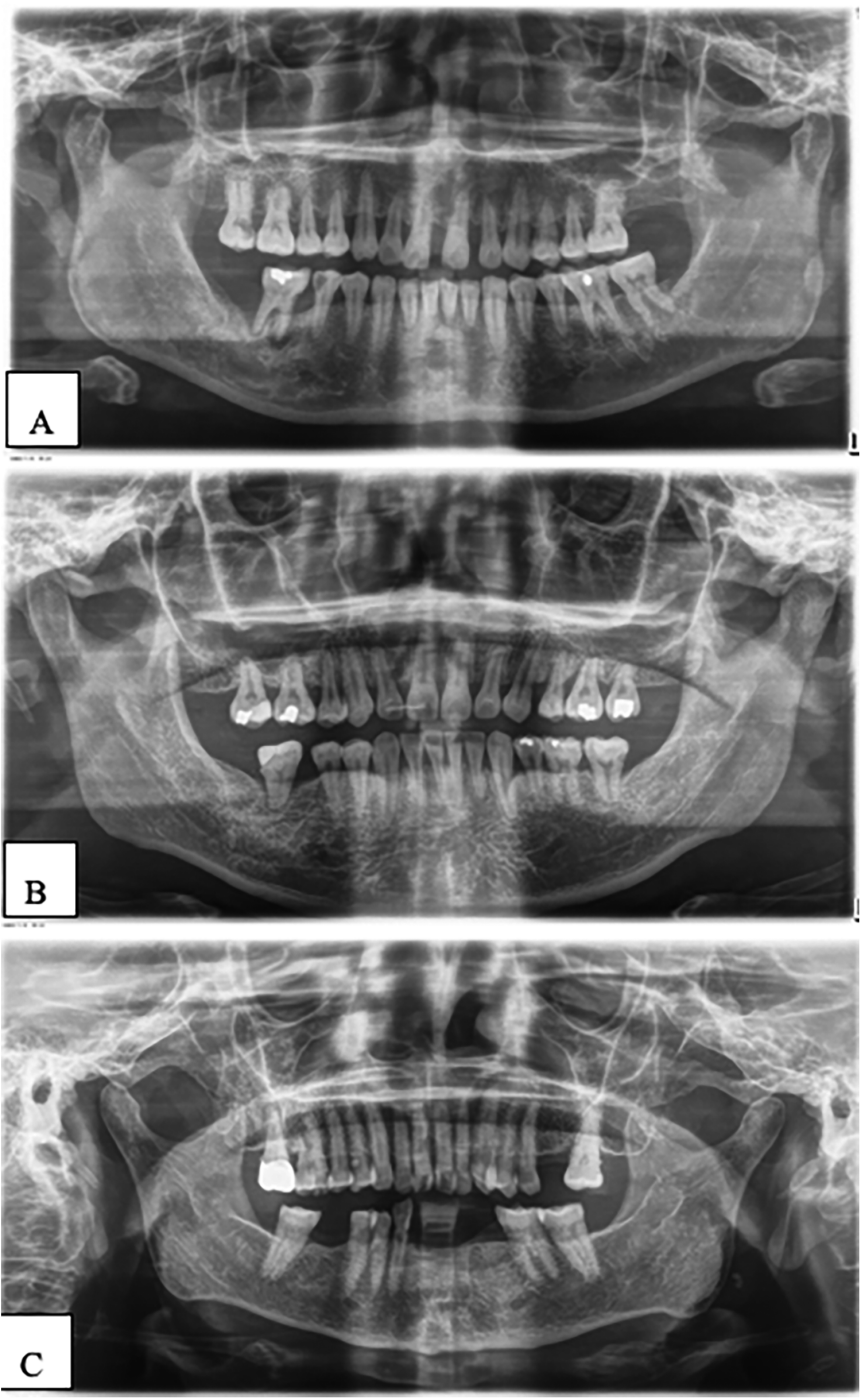
**(A–C)** Examples of panoramic radiographs captured using the ORTHOPHOS XG device (Sirona, Bensheim, Germany).

**Figure 6:**
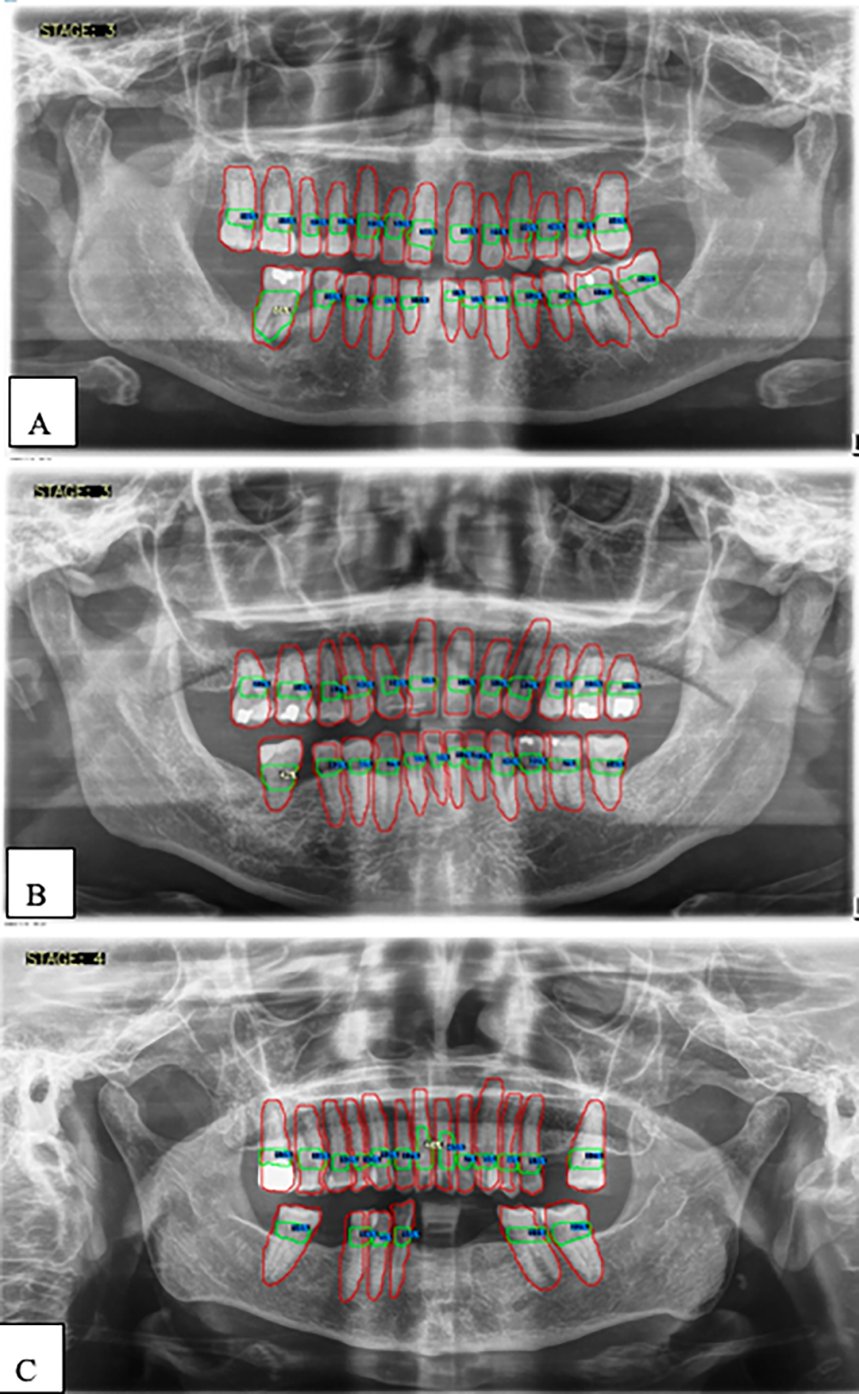
**(A–C)** Examples illustrating the percentages of alveolar bone loss for each tooth. Red lines indicate individual teeth, green lines represent the space between the cemento-enamel junction (CEJ) and the alveolar bone level, blue numbers denote the percentage of bone loss for each tooth, and white numbers highlight the greatest bone loss observed.

Figures 5, 6 show panoramic radiographs captured with the ORTHOPHOS XG device (Sirona, Bensheim, Germany) and illustrate the percentage of alveolar bone loss for each tooth compared to the periodontal prognosis categories defined by the Thai Association of Periodontology, aiding in individual prognosis determination.

## Discussion

This study aims to develop an innovative AI-driven model using state-of-the-art CNNs to analyze the percentage of alveolar bone loss assessment and given the individual periodontal prognosis from the panoramic radiographs. The total dataset, consisting of 2,000 panoramic radiographs diagnosed with periodontitis. The primary outcome variables involve the performance of these segmentation tasks, which are critical for diagnosing periodontal disease and assessing its prognosis based on radiographic features. The performance metrics of two AI models developed for analyzing panoramic radiographs: one for segmenting teeth and the other for segmenting the cemento-enamel junction (CEJ) and alveolar bone levels. This study presents precision measures, with the CEJ and bone level segmentation model outperforming the teeth segmentation model (0.90 vs. 0.80). The F1 Score, reflecting the harmonic mean of precision and sensitivity, was 0.80 for teeth segmentation and 0.90 for the CEJ and bone level segmentation, indicating balanced performance. Sensitivity was perfect (1.0) for the CEJ and bone level model, while the teeth model achieved 0.90. Specificity was 0.96 for the teeth segmentation model and 0.98 for the CEJ and bone level model. Accuracy was higher for the CEJ and bone level model (0.98) compared to the teeth model (0.97), and the Mean Average Precision (mAP50) further emphasized the superiority of the CEJ and bone level model (0.995 vs. 0.92). Overall, the CEJ and bone level segmentation model demonstrated superior performance across all metrics. These results are comparable to, and in some cases exceed, those reported in previous studies (16). Notably, the CEJ and alveolar bone level segmentation model outperformed the teeth segmentation model across all metrics, achieving precision, recall, and F1-Score values of 0.90, 1.0, and 0.90, respectively. This high performance underscores the model's ability to accurately detect key periodontal structures, which is crucial for assessing bone loss and predicting disease progression in accordance with the periodontal prognosis categories defined by the Thai Association of Periodontology (13). Furthermore, the model has the potential to reduce the workload of dental professionals by providing consistent and reliable periodontal assessments (33).

The CEJ and alveolar bone level segmentation model's perfect recall of 1.0 is particularly noteworthy, indicating its robustness in identifying true positives and minimizing missed diagnoses in clinical practice. This capability ensures comprehensive periodontal assessments by accurately identifying all instances of alveolar bone loss in the test set (34). Additionally, the high F1-Score of 0.90 demonstrates a well-balanced performance between precision and recall, highlighting the model's ability to maintain accuracy without compromising sensitivity. Compared to similar studies, this research shows superior performance in both teeth segmentation and CEJ and alveolar bone level segmentation. The teeth segmentation model's precision, recall, and F1-Score of 0.80, 0.90, and 0.80, respectively, are in line with those reported for deep learning-based models by Krois et al. and Bayrakdar et al. (34, 35). However, the CEJ and alveolar bone level segmentation model in this study surpasses previous models, achieving precision, recall, and F1-Score values of 0.90, 1.0, and 0.90, respectively. This highlights the model's robustness and its ability to accurately detect critical periodontal structures, thereby reducing the risk of missed diagnoses.

In comparison with other AI implementations in dentistry, such as those reviewed by Schwendicke et al. and Topol (14, 15), the model developed in this study offers enhanced diagnostic capabilities. Its targeted focus on periodontal structures contributes to its superior performance. AI's potential to reduce subjectivity in diagnostic processes is a significant advantage. Schwendicke et al. (14) discussed how AI could standardize diagnostic outcomes by minimizing human error, which is often a factor in manual assessments by practitioners. Additionally, the integration of AI with telehealth platforms, as observed during the COVID-19 pandemic, underscores the role of technology in enhancing accessibility and consistency in patient care (36). Consequently, the application of AI in routine dental practice could improve consistency and reliability in periodontal assessments, ultimately leading to better patient outcomes.

The demographic analysis of the dataset, presented in Table 2, shows a balanced representation of male and female patients, with a slight predominance of females (1,177 females vs. 823 males). The mean age of participants was 47.04 years for males and 45.27 years for females, aligning with the typical age range associated with higher susceptibility to periodontal diseases. This enhances the relevance of the study's findings to real-world clinical scenarios (9).

While previous studies have focused on using AI to detect periodontal bone loss from panoramic radiographs, this study is the first to develop an AI-driven model using state-of-the-art CNNs to analyze the percentage of alveolar bone loss and provide individualized periodontal prognoses from panoramic radiographs (33–35, 37–48). This innovative approach not only enhances diagnostic consistency but also represents a significant advancement in the identification and management of periodontal conditions. As AI continues to evolve, this model represents a crucial step toward fully integrating AI technologies into routine dental practice, ultimately improving patient outcomes.

One of the most significant issues highlighted by this study is the high prevalence of periodontal disease, particularly in developing countries such as Thailand. According to the Bureau of Dental Health, Department of Health, the National Oral Health Survey conducted every five years indicates that the prevalence of periodontitis in Thailand is higher than the global average reported by the World Health Organization (WHO) (49). The Global Burden of Disease Study 2017 reported that severe periodontal disease affects 19% of adults worldwide, over 1 billion people, making it the 11th most prevalent disease globally (5, 6). However, the latest survey in 2023 revealed that 48.7% of older patients in Thailand suffer from periodontitis, an increase from 36.3% in the previous survey. The highest prevalence was found in the Northern Region at 58.4%, followed by the Southern Region at 56.7%, the North-Eastern Region at 47.1%, and the Central Region at 42.3% (49).

These alarming the urgent need for improved disease prevention and highlight the importance of periodontal health. The AI models developed in this study offer a promising solution by providing quicker, less labor-intensive, and more precise alternatives to current approaches. This is crucial for treatment planning, helping dentists decide on the management of periodontal disease, including whether to retain or extract affected teeth immediately after uploading the panoramic radiograph into the developed AI software for each patient. If the Ministry of Public Health, as the central policy-maker, prioritizes this critical issue and supports the deployment of our AI model nationwide and globally, we could significantly reduce the prevalence of periodontal disease, thereby improving the overall quality of life for the population.

This study has several limitations, the most notable being its reliance on data from a single institution, Fang Hospital in Chiang Mai, Thailand. The use of data from a single center may introduce biases related to patient demographics, regional healthcare practices, and imaging techniques, which could limit the generalizability of the findings. Additionally, the study exclusively utilized high-quality panoramic radiographs from the SIDEXIS Next Generation Program (Sirona, Bensheim, Germany). While this ensured consistent image quality, it may restrict the model's applicability to radiographs obtained from other systems, as differences in resolution between x-ray devices can affect the performance of the developed AI software, which currently cannot account for such variations. Furthermore, low-quality images caused by patient movement, incorrect positioning, or rare bone morphologies were excluded, potentially leading to an underrepresentation of more challenging cases. In comparison with existing studies (14, 15, 23, 33–35, 37–48), our results highlight the superior performance of our AI models in detecting periodontal alveolar bone loss compared to previous AI applications in dentistry. Future research should aim to validate these models across multiple centers, integrate other imaging modalities, and explore real-time clinical applications to refine predictive capabilities, particularly for suboptimal images.

## Conclusion

This AI model establishes a state-of-the-art approach for assessing alveolar bone loss and identifying individual periodontal prognoses. It offers a promising solution by providing faster, less labor-intensive, and more accurate alternatives to existing methods.

## Data Availability

The data analyzed in this study is subject to the following licenses/restrictions: You can request. Requests to access these datasets should be directed to Jarupat Jundaeng, sukijarupat@gmail.com.
